# Novel Mesenchymal Stem Cell Spheroids with Enhanced Stem Cell Characteristics and Bone Regeneration Ability

**DOI:** 10.1093/stcltm/szab030

**Published:** 2022-03-10

**Authors:** Yumi Ohori-Morita, Kunimichi Niibe, Phoonsuk Limraksasin, Praphawi Nattasit, Xinchao Miao, Masahiro Yamada, Yo Mabuchi, Yumi Matsuzaki, Hiroshi Egusa

**Affiliations:** Division of Molecular and Regenerative Prosthodontics, Tohoku University Graduate School of Dentistry, 4-1 Seiryo-machi, Aoba-ku, Sendai, Miyagi 980-8575, Japan; Division of Molecular and Regenerative Prosthodontics, Tohoku University Graduate School of Dentistry, 4-1 Seiryo-machi, Aoba-ku, Sendai, Miyagi 980-8575, Japan; Division of Molecular and Regenerative Prosthodontics, Tohoku University Graduate School of Dentistry, 4-1 Seiryo-machi, Aoba-ku, Sendai, Miyagi 980-8575, Japan; Division of Molecular and Regenerative Prosthodontics, Tohoku University Graduate School of Dentistry, 4-1 Seiryo-machi, Aoba-ku, Sendai, Miyagi 980-8575, Japan; Division of Molecular and Regenerative Prosthodontics, Tohoku University Graduate School of Dentistry, 4-1 Seiryo-machi, Aoba-ku, Sendai, Miyagi 980-8575, Japan; Division of Molecular and Regenerative Prosthodontics, Tohoku University Graduate School of Dentistry, 4-1 Seiryo-machi, Aoba-ku, Sendai, Miyagi 980-8575, Japan; Department of Biochemistry and Biophysics, Graduate School of Medical and Dental Sciences, Tokyo Medical and Dental University, 1-5-45 Yushima, Bunkyo-ku, Tokyo 113-8510, Japan; Department of Life Science, Faculty of Medicine, Shimane University, 89-1 Enya-cho, Izumo, Shimane 693-8501, Japan; Division of Molecular and Regenerative Prosthodontics, Tohoku University Graduate School of Dentistry, 4-1 Seiryo-machi, Aoba-ku, Sendai, Miyagi 980-8575, Japan; Center for Advanced Stem Cell and Regenerative Research, Tohoku University Graduate School of Dentistry, Sendai, Miyagi 980-8575, Japan

**Keywords:** bone regeneration, immunomodulatory ability, mesenchymal stem cells, neurosphere culture medium, shaking culture

## Abstract

Mesenchymal stem cells (MSCs) exhibit self-renewal, multi-lineage differentiation potential and immunomodulatory properties, and are promising candidates for cellular therapy of various tissues. Despite the effective function of MSCs, the gradual loss of stem cell characteristics that occurs with repeated passages may significantly limit their therapeutic potential. A novel 3D shaking method was previously established to generate MSC spheroids in growth medium (GM-spheroids) and successfully maintain the multipotency of expanded MSCs, yet the expression of MSC-related genes was still low. In this study, we used a neurosphere culture technique to optimize the shaking culture method using human bone marrow-derived MSCs (BM-MSCs). MSC spheroids generated in neurosphere medium (NM-spheroids) maintained high expression of MSC-related genes during 3 weeks of prolonged shaking culture. Moreover, NM-spheroids generated from expanded MSCs showed high viability, upregulation of MSC-related and immune-related genes, and recovery of differentiation potential in vitro. Expanded adherent MSCs, GM-spheroids, and NM-spheroids were transplanted into a rat femur bone defect model to investigate their therapeutic potential in bone repair. Adherent MSCs and GM-spheroids showed delayed bone healing. In contrast, NM-spheroids showed high transplantation efficiency and enhanced bone regeneration. These data suggest that NM-spheroids generated using modified neurosphere culture conditions under continuous shaking recovered their stem cell characteristics in vitro and enhanced bone regeneration in vivo. Therefore, NM-spheroids should have great clinical potential for bone and tissue regenerative therapies as a stem cell-based biomaterial therapy.

## Graphical Abstract

**Graphical Abstract:**
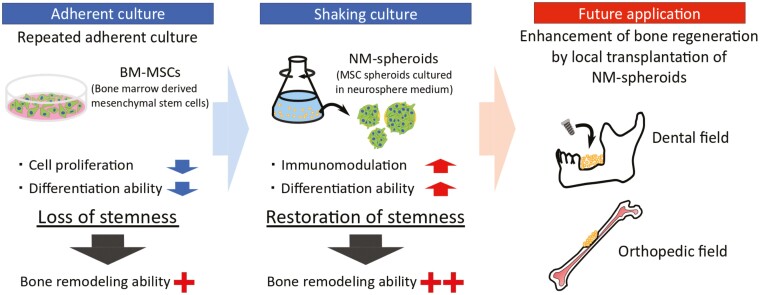

**Significance Statement**

Mesenchymal stem cells (MSCs) are conventionally expanded by adherent culture, which has been reported to gradually change their stem cell characteristics and alter their therapeutic potential. As an alternative culture system, we developed a 3D shaking method using a neurosphere medium to generate MSC spheroids from expanded MSCs. The MSC spheroids maintained high expression of stem cell genes and retained, even recovered, their multipotency during prolonged shaking culture, which suggests long-term maintenance of stem cell characteristics. Moreover, transplantation of MSC spheroids in a rat femur defect model demonstrated high transplantation efficiency and enhanced bone regeneration. Thus, the novel 3D shaking culture of MSCs represents a promising method for regeneration therapies.

## Introduction

Mesenchymal stem cells (MSCs) exhibit self-renewal and multi-lineage differentiation ability.^1^ Owing to their unique biological properties and functions, MSCs are considered promising candidates for cellular therapy for various disorders. In particular, bone marrow-derived MSCs (BM-MSCs) have been used for bone tissue regeneration in orthopedic and dental medicine,^2,3^ and transplantation of MSCs has been shown to be beneficial for the treatment of fixed fractures with delayed union or nonunion.^4^ However, clinical applications of MSC transplantation have shown inconsistent results.^5^

One of the possible causes for inconsistent results is the variability of donors with regard to healing potential.^6-8^ In addition, the isolation efficiency of MSCs varies among donors because of the lack of a definitive isolation technique for MSCs.^9^ During isolation of MSCs from patient bone marrow, various types of cells grow as plastic-adherent cells, which is unavoidable. Therefore, conventional methods are still insufficient to isolate pure MSCs. To overcome these issues, recent studies have attempted to identify MSCs by combining several surface markers. Low-affinity nerve growth factor receptor (LNGFR, known as CD271)^+^ and thymus cell antigen-1 (THY-1, known as CD90)^+^ human BM-MSCs exhibit high differentiation potential and functional properties, thereby identifying them as an enriched subpopulation of human BM-MSCs.^9,10^ In addition, THY-1^+^ MSCs have been reported to promote in vitro osteogenesis and in vivo bone formation^11^; therefore, LNGFR^+^/THY-1^+^ BM-MSCs may facilitate MSC transplantation in bone regeneration.

Another possible problem is the expansion of MSCs, which conventionally expand as adherent monolayers in vitro; several studies have reported phenotypic changes and replicative senescence of MSCs after repeated culture and passages.^12,13^ In particular, expanded MSCs show altered differentiation potential^14,15^ and immunomodulatory capacity.^16,17^ These changes impair the stem cell characteristics of MSCs, which may significantly limit their therapeutic potential.

Three-dimensional (3D) culture of stem cells has gained attention as an alternative to conventional adherent culture. Stem cells form 3D cell aggregates, often referred to as cell spheroids, via self-assembly in non-adherent conditions. These 3D multicellular spheroids are thought to more closely resemble the native microenvironment by providing greater cell-cell and cell-matrix interactions than conventional monolayer cultures.^18,19^ Indeed, several studies have reported advantages of MSC spheroids, such as enhanced pluripotency, higher differentiation potential, and enhanced anti-inflammatory properties.^18,20-22^ In addition, MSC spheroids, which can be used as a scaffold-free biomaterial, have been reported to have enhanced osteogenic differentiation and to promote in vivo bone regeneration.^23,24^ However, most experiments were only able to maintain spheroids in a culture system from several hours to 2 weeks at the longest.

We previously established a novel 3D shaking culture method that successfully maintained LNGFR^+^/THY-1^+^ human MSC spheroids with high viability and multipotency for up to 1 month.^25^ The spheroids maintained their 3D rounded shape after attaching to a plastic plate and provided undifferentiated MSCs continuously in vitro. Despite the great potential of MSC spheroids generated by this method, several MSC- or neural crest stem cell (NCSC)-related genes, such as *NESTIN* (*NES*) and *SOX2*, showed deficient expression.^25^ Thus, in this study, we attempted to optimize the culture conditions of our shaking method for MSCs, taking a cue from neurosphere culture.

Neurosphere culture is a specific technique for isolating and maintaining NCSCs and progenitor cells by forming cell spheroids.^26^ Several studies have reported that neurospheres generated from dental tissue-derived stem cells, which are known to originate from the neural crest, show multi-lineage differentiation toward mesenchymal lineages.^27,28^ Moreover, Peng et al demonstrated that human umbilical cord-derived MSCs generate neurospheres with features of both MSCs and NCSCs.^29^ Therefore, we expected that enriched human MSCs cultured in neurosphere culture conditions would maintain both NCSC and MSC phenotypes, with enhanced stem cell characteristics.

Based on this background, we hypothesize that a modified 3D shaking culture method in neurosphere culture conditions will enhance stem cell characteristics and promote the therapeutic potential of enriched human MSCs for bone regeneration. The aim of this study was to investigate the characteristics of MSC spheroids generated by a 3D shaking culture method modified using neurosphere culture conditions to examine their therapeutic potential in bone regeneration.

## Materials and Methods

### BM-MSC Adherent Culture

Human BM-MSCs of an 18-year-old male (donor 1; Lonza, Basel, Switzerland), a 22-year-old male (donor 2; AllCells, Alameda, CA, USA), and a 24-year-old male (donor 3; Lonza) were purchased. MSCs were enriched with PI^−^/LNGFR^+^/THY-1^+^ markers and maintained in growth medium (GM) consisting of Dulbecco’s modified Eagle’s medium (DMEM) with 4.5 g/L glucose without sodium pyruvate (Nacalai Tesque, Kyoto, Japan), 20% fetal bovine serum (FBS) (GE Healthcare Hyclone, Logan, UT, USA), 1% penicillin-streptomycin (Wako, Osaka, Japan), 10 mM HEPES (Dojindo, Kumamoto, Japan), and 10 ng/mL recombinant human basic fibroblast growth factor (bFGF) (Wako).^10^ MSCs were cultured and incubated at 37°C with 5% CO_2_ at a cell density of 3 × 10^4^ cells/mL in 10-cm culture dishes (Greiner Bio-One, Kremsmunster, Australia). We prepared MSCs with 5-9 passages as low-passage adherent MSCs and MSCs with 15-18 passages as high-passage adherent MSCs.

### 3D Shaking Culture of BM-MSCs

Human BM-MSCs were seeded at 5 × 10^5^ cells/mL (1 × 10^7^ cells/20 mL/flask) in a culture flask (Corning, NY, USA) in GM or neurosphere medium (NM) consisting of advanced DMEM (Gibco, Waltham, MA, USA), 1% penicillin-streptomycin (Wako), 1% l-glutamine (Wako), 10 mM HEPES (Dojindo), 20 ng/mL recombinant human epidermal growth factor (EGF) (Wako), 20 ng/mL recombinant human bFGF (Wako), 2% N-2 (Gibco), and 2% B27 (Gibco). Cells were cultured in a bio-shaker (BR-40LF; Taitec, Saitama, Japan) at 37°C under 5% CO_2_, with shaking at 85-95 rpm and 40 mm amplitude. Half of the medium was renewed every 3-4 days. For experiments using plated MSC spheroids, the spheroids were collected and plated onto tissue culture plates in GM. Cells were allowed to grow out from plated spheroids for 3-7 days with medium exchange every 3-4 days.

### Reverse-Transcription Polymerase Chain Reaction (RT-PCR) Analysis

Total RNA was extracted from the cells using TRIzol reagent (Invitrogen). mRNA was purified using an RNeasy Mini Kit (Qiagen, Hilden, Germany) and quantified using a spectrophotometer (NanoDrop One; Thermo Fisher Scientific). After treatment with DNase I (Invitrogen), cDNA was synthesized using a thermal cycler (GeneAtlas G; Astec, Fukuoka, Japan) from 1 µg of mRNA on a Reverse Transcription System (Promega) according to the manufacturer’s instructions.

For real-time RT-PCR analysis, a SYBR Green assay was performed using Thunderbird SYBR qPCR Mix (Toyobo, Osaka, Japan) on a StepOnePlus real-time PCR system (Thermo Fisher Scientific). Target gene expression was quantitatively measured using the comparative Ct method.^30^ The primer pairs used are listed in Supplementary Table S1.

### PCR Array Analysis

Total RNA was extracted from the cells using TRIzol reagent (Invitrogen). mRNA was purified using an RNeasy Mini Kit (Qiagen) and quantified using a spectrophotometer (NanoDrop One; Thermo Fisher Scientific). cDNA was synthesized using the RT^2^ First Strand Kit (Qiagen) according to the manufacturer’s instructions. The human cell cycle RT^2^ Profiler PCR Array Kit (Qiagen), which comprises 84 cell cycle-related genes, was used to investigate the expression profiles in adherent MSCs and spheroids. PCR was performed using RT^2^ SYBR Green ROX qPCR Mastermix (Qiagen) and a StepOnePlus real-time PCR system (Thermo Fisher Scientific). The array data were normalized with a panel of internal control genes: *ACTB*, *B2M*, *GAPDH*, *HPRT1*, and *RPLP0*. Data were analyzed using a web-based software package (Qiagen).

### Transplantation of MSCs to Rat Femurs

The animal experiments conducted in this study were approved by the Animal Research and Care Committee of Tohoku University (approval no. 2018DnA-002, 2019DnA-052-02). High-passage adherent MSCs were prepared for cell transplantation. Adherent cultured cells were harvested and re-suspended in GM to a concentration of 1 × 10^6^ cells/mL. A commercial type I collagen-based dressing sponge for dental surgery (Collaplug; Integra Life Sciences, Plainsboro, NJ, USA) was used as the vehicle for MSC transplantation. The sponge was shaped into a semicircular column (5 × 5 × 2 mm), and a rectangular portion was formed into a double fold. The double-folded sponges were placed on a 48-well culture plate, ensuring that the well bottom was completely covered. Subsequently, 1 mL of cell suspension or MSC spheroids was seeded on the sponge and the plate was incubated at 37°C with 5% CO_2_ for 12 hours. Because spheroids strongly aggregated, equal dissociation of the spheroids into single cells for obtaining an accurate cell count was technically difficult. Therefore, the amount of DNA in adherent cells and spheroids was measured in advance of transplantation to equalize the number of cells (1 × 10^6^ cells) in each transplant sample.

For this experiment, 11-week-old Sprague-Dawley (SD) rats (Clea Japan, Tokyo, Japan) were used following an established protocol with modification.^31^ The animals were randomly assigned to six groups: defects treated with collagen sponge containing adherent MSCs for 3 days (*n* = 3), defects treated with collagen sponge containing NM-spheroids for 3 days (*n* = 3), defects treated with collagen sponge without cells for 3 weeks (*n* = 5), defects treated with collagen sponge containing adherent MSCs for 3 weeks (*n* = 5), defects treated with collagen sponge containing GM-spheroids for 3 weeks (*n* = 5) and defects treated with collagen sponge containing NM-spheroids for 3 weeks (*n* = 8). Briefly, the animals were anesthetized with 2% isoflurane (Mylan, Canonsburg, PA, USA), and a large rectangular segmental resection (5 × 3 mm) was made under irrigation in the center part of the left femur cortical bone with a round steel burr. Rats were treated with 5 mg/kg carprofen (Zoetis Japan, Tokyo, Japan) for postoperative analgesia and 10 mg/kg/day cyclosporine (LC Laboratories, Woburn, MA, USA) to induce immunosuppression. At 3-day and 3-week post-surgery, the rats were sacrificed by cervical dislocation under isoflurane anesthesia, and the left femur was collected for micro-computed tomography (micro-CT) and histological examination.

### Statistical Analysis

One-way analysis of variance (ANOVA) was used to assess differences among multiple experimental groups, and when appropriate, the Tukey-Kramer test and Dunnett’s test were used for post hoc tests. For comparisons between two groups, Student’s *t* test was used. *P* < .05 was considered statistically significant. Statistical analysis was performed using IBM SPSS Statistics 21 statistical software (IBM Japan, Ltd., Tokyo, Japan).

Methods for induction of mesenchymal lineage and neural crest lineage, cell viability, DNA quantification, senescence-associated β-galactosidase (SA-β-gal) assay, histological and immunocytochemistry assay, and micro-CT analysis are provided in the Supplementary information.

## Results

### Phenotypic Changes in Human BM-MSCs after Long-term Adherent Culture

**Figure 1.**
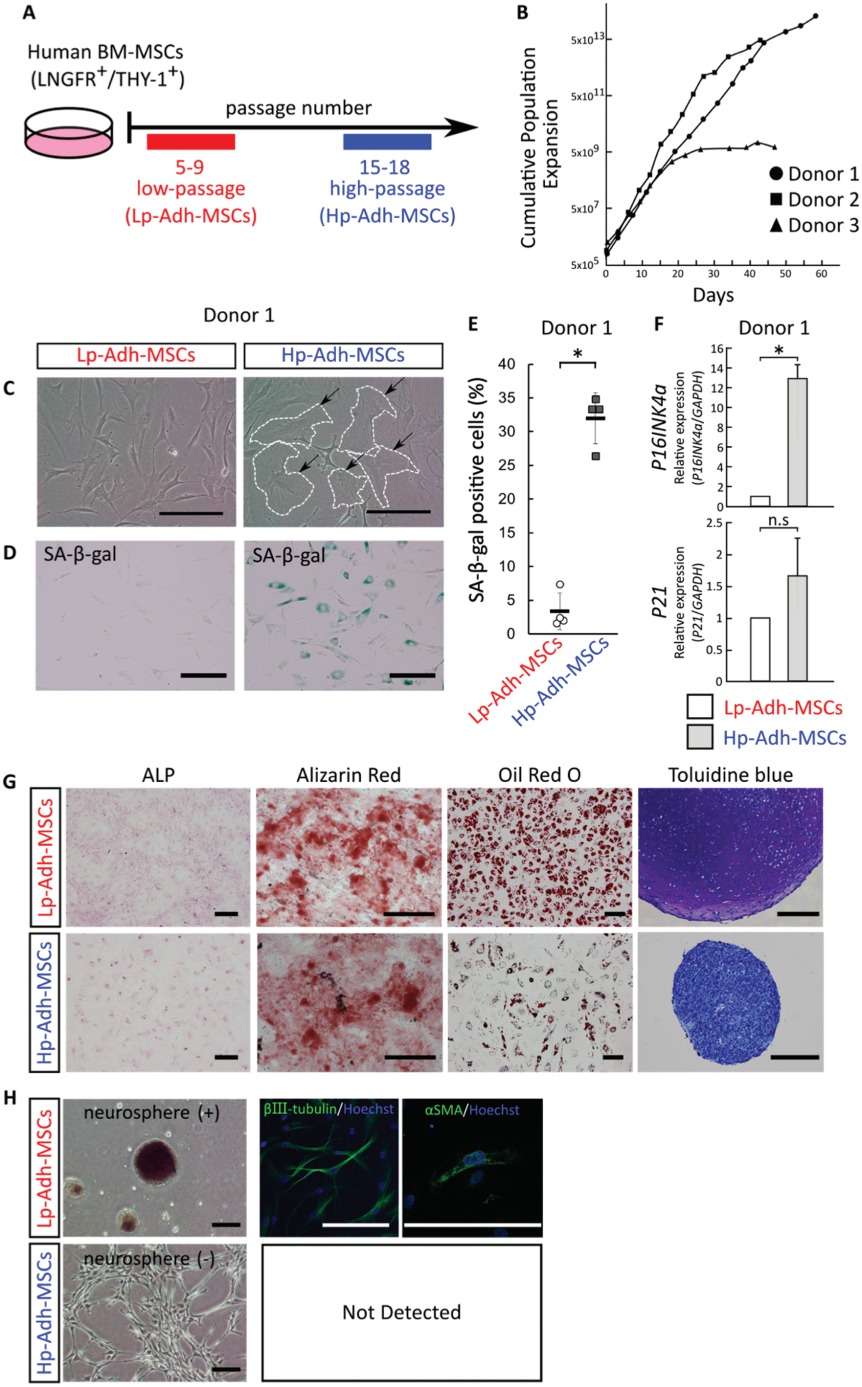
Phenotypic changes in human BM-MSCs after long-term adherent culture. (A) Schema of a conventional adherent culture system. Human BM-MSCs were cultured for a short period [passage 5-9: low-passage adherent MSCs (Lp-Adh-MSCs)] and a long period [passage 15-18: high-passage adherent MSCs (Hp-Adh-MSCs)]. (B) Growth profiles of human BM-MSCs from 3 donors as a cumulative population expansion. (C) Phase-contrast images of Lp-Adh-MSCs and Hp-Adh-MSCs (donor 1). Notes: Arrowhead: location of enlarged cells; dotted line: outline of enlarged cells. Scale bars = 200 µm. (D) SA-β-gal assay of Lp-Adh-MSCs and Hp-Adh-MSCs (donor 1). Scale bars = 200 µm. (E) Quantitative analysis of SA-β-gal-positive cells (mean ± SD, *n* = 4; *P* < .05, Student’s *t* test). (F) Relative expression of *P16INK4a* and *P21* determined by real-time RT-PCR (mean ± SD, *n* = 3; *P* < .05, n.s, not significant, Student’s *t* test). (G) Mesenchymal lineages derived from Lp-Adh-MSCs and Hp-Adh-MSCs. Osteocytes, adipocytes, and chondrocytes were identified by staining with ALP, Alizarin Red, Oil Red O, and Toluidine blue, respectively. Scale bars = 200 µm. (H) Induction of BM-MSCs cell aggregation in neurosphere conditions. Differentiation toward neural cells and smooth muscle cells was determined by immunocytochemistry for βш-tubulin and αSMA. Cells were counterstained with Hoechst to reveal the nuclei. Not detected: the test could not be performed. Scale bars = 200 µm. Abbreviations: BM-MSCs, bone marrow-derived mesenchymal stem cells; RT-PCR, reverse-transcription polymerase chain reaction; αSMA, alpha-smooth muscle actin.

Human BM-MSCs were cultured as adherent monolayers and continuously passaged until the cell expansion limit (Fig. 1A). The cell proliferation rate declined for cells from all three donors after repeated passages (Fig. 1B). Low-passage (Lp)-adherent MSCs (Lp-Adh-MSCs) retained their spindle shape; however, high-passage (Hp)-adherent MSCs (Hp-Adh-MSCs) appeared enlarged with a changed morphology (Fig. 1C; Supplementary Fig. S1A, dotted lines). Hp-Adh-MSCs showed a greater increase in SA-β-gal-positive cells than Lp-Adh-MSCs (mean ± SD, *n* = 4, *P* < .05, Fig. 1D, 1E; Supplementary Fig. S1B, S1C). Cellular senescence is triggered by DNA damage. The gene expression of the cyclin-dependent kinase inhibitors *P16INK4a* and *P21* was increased in Hp-Adh-MSCs compared with Lp-Adh-MSCs in cells from all donors and was significantly increased in cells from donors 2 and 3, respectively (Fig. 1F; Supplementary Fig. S1D). In addition, karyotyping analysis did not show chromosomal abnormalities in Hp-Adh-MSCs cells (data not shown). These results indicate that repeated passaging induced cellular senescence in human-enriched BM-MSCs.

Cells from donor 1 maintained stable proliferation until 18 passages (Fig. 1B). The representative differentiation assay for mesenchymal lineages of donor 1 showed that Lp-Adh-MSCs differentiated toward ALP^+^ and Alizarin Red^+^ osteocytes, Oil Red O^+^ adipocytes, and Toluidine blue^+^ chondrocytes, whereas Hp-Adh-MSCs showed impaired adipogenesis (decreased formation of adipocyte lipid droplets) and chondrogenesis (without Toluidine blue staining in purple for cartilage matrix) (Fig. 1G). MSCs have been reported to have the potential to differentiate into neural crest lineages^32,33^ through culture in serum-free NM for 14 days.^32^ Following this protocol, Lp-Adh-MSCs formed neurospheres, and βш-tubulin^+^ neuron-like cells and αSMA^+^ smooth muscle-like cells were observed (Fig. 1H). In contrast, Hp-Adh-MSCs attached to the culture dish and failed to form neurospheres (Fig. 1H). These results suggest that human BM-MSCs lose their multipotency after prolonged adherent culture, similar to previous reports.^12,15^

### Optimization and Characterization of MSC Spheroids with NM under 3D Shaking Culture

In this study, we applied 3D shaking culture method^25^ to Lp-Adh-MSCs in NM to fabricate spheroids (LpMSC-NM-spheroids), and cultured the cells for up to 4 weeks (Supplementary Fig. S2A). Lp-Adh-MSC-derived GM-spheroids (LpMSC-GM-spheroids) cultured for 4 weeks were prepared as a control. LpMSC-NM-spheroids were observed after 3 days of shaking culture and were found to maintain their well-organized circular shape, even after culturing for 4 weeks (Supplementary Fig. S2B). LpMSC-NM-spheroids had a diameter of ~1200 µm, and the diameter did not significantly change during prolonged shaking culture (mean ± SD, *n* = 10, Supplementary Fig. S2C). LpMSC-NM-spheroids strongly aggregated and could not be dissociated by pipetting or trypsinization. Thus, total DNA was measured to investigate cell proliferation. The DNA quantity was significantly different between Lp-Adh-MSCs and LpMSC-NM-spheroids cultured for 3 weeks (Supplementary Fig. S2D). However, the DNA quantity did not increase in LpMSC-NM-spheroids, even though they contained Lp-Adh-MSC-derived cells with high proliferation ability. In contrast, the neural crest markers *SOX9*, *SNAI2* (*SLUG*), and *NESTIN* (*NES*), the MSC marker vascular cell adhesion molecule-1 (*VCAM-1*), and the stem cell marker *SOX2* showed higher expression in LpMSC-NM-spheroids than in both Lp-Adh-MSCs and LpMSC-GM-spheroids by quantitative analysis and maintained stable gene expression during culturing from 1 to 4 weeks (Supplementary Fig. S2E-S2G).

**Figure 2.**
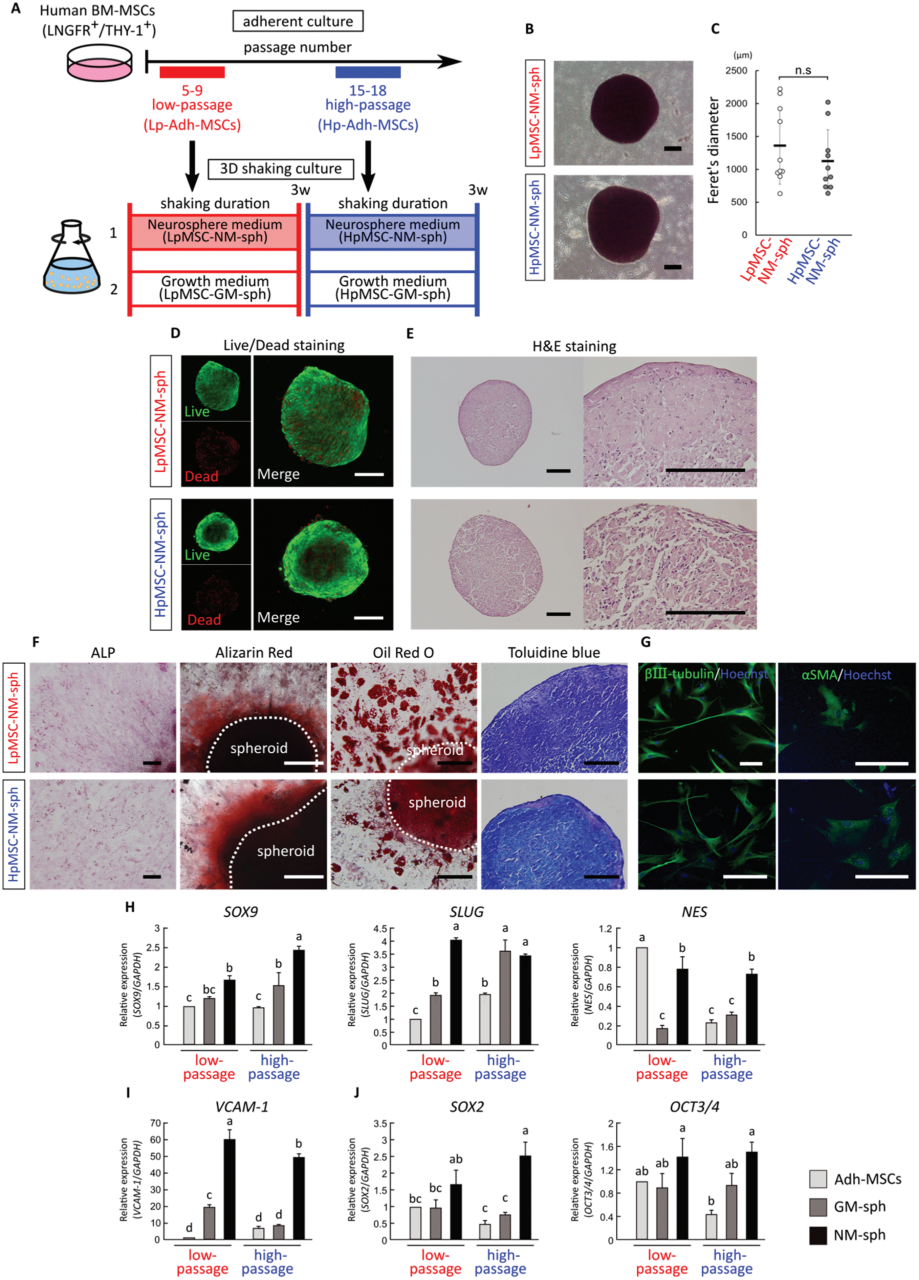
Characterization of NM-spheroids. (A) Schema of a 3D shaking culture system with cells derived from low-passage adherent MSCs (Lp-Adh-MSCs) and high-passage adherent MSCs (Hp-Adh-MSCs). (B, C) Phase-contrast images of Lp-Adh-MSC-derived NM-spheroids (LpMSC-NM-sph) and Hp-Adh-MSC-derived NM-spheroids (HpMSC-NM-sph) cultured for 3 weeks and their Feret’s diameter. Scale bars = 200 µm (mean ± SD, *n* = 10; *P* < .05, Student’s *t* test). (D) 3D reconstruction images of live/dead staining of NM-spheroids. Scale bars = 200 µm. (E) H&E staining of LpMSC-NM-sph and HpMSC-NM-sph. Scale bars = 200 µm. (F) Mesenchymal lineages derived from LpMSC-NM-sph and HpMSC-NM-sph. Osteocytes, adipocytes, and chondrocytes were identified by staining with ALP, Alizarin Red, Oil Red O, and Toluidine blue, respectively. Scale bars = 200 µm. Dotted line: outline of NM-spheroids. (G) Differentiation toward neural cells and smooth muscle cells, determined by immunocytochemistry for βш-tubulin and αSMA. Cells were counterstained with Hoechst to reveal the nuclei. Scale bars = 200 µm. (H-J) Relative expression of (H) neural crest markers (*SOX9*, *SLUG*, *NES*), (I) MSC marker (*VCAM-1*), and (J) stem cell markers (*SOX2*, *OCT3/4*). *GAPDH* expression was used as an internal control (mean ± SD, *n* = 3; different letters indicate significant difference, *P* < .05, ANOVA with Tukey’s multiple comparison test). Adh-MSCs: Adherent MSCs (sorted by LNGFR and THY-1), GM-sph: 3D spheroids cultured with GM, NM-sph: 3D spheroids cultured with NM. Abbreviations: GM, growth medium; MSCs, mesenchymal stem cells; NM, neurosphere medium.

Because LpMSC-NM-spheroids cultured for 3 weeks showed high expression of neural crest, MSC, and stem cell markers, Hp-Adh-MSC-derived spheroids with a 3-week culture were prepared for further experiments (Fig. 2A). The LpMSC-NM-spheroids and Hp-Adh-MSC-derived NM-spheroids (HpMSC-NM-spheroids) showed a circular shape (Fig. 2B) with no significant difference in their sizes (mean ± SD, *n* = 10, Fig. 2C). Most cells in both LpMSC- and HpMSC-NM-spheroids were alive, suggesting that the long adherent culture duration did not markedly affect cell viability (Fig. 2D). The inner cells of the spheroids formed a rounded shape, and the cells in the surface region showed a spindle shape and formed cell layers (Fig. 2E).

NM-spheroids were able to attach to the adherent culture dish, and fibroblastic cells migrated from the spheroids. These migrated cells showed differentiation potential toward osteocyte and adipocyte lineages (Fig. 2F). For chondrogenic induction, NM-spheroids were directly incubated in chondrogenic induction medium, and only the peripheral region of the spheroids was stained with Toluidine blue (Fig. 2F). NM-spheroids retained their 3D shape after re-seeding on plastic culture dishes, similar to GM-spheroids.^25^ This phenomenon indicates that the cells in the spheroids adhered strongly to their neighboring cells or extracellular matrix (ECM), which may have limited the penetration of the induction medium into the inner region of the spheroids. In addition, the differentiation to the neural crest lineage was impaired for Hp-Adh-MSCs (Fig. 1H). Notably, both LpMSC- and HpMSC-NM-spheroids showed βIII-tubulin^+^-neuron-like cells and αSMA^+^-smooth muscle-like cells (Fig. 2G). These results indicate that NM-spheroids culture restored adipogenic and neurogenic differentiation abilities in Hp-Adh-MSCs.

We evaluated the gene expression profiles of NM-spheroids from Lp-Adh-MSCs and Hp-Adh-MSCs cultured for 3 weeks. With regard to neural crest markers, both LpMSC- and HpMSC-NM-spheroids showed high expression of *SOX9*, *SLUG*, and *NES* (Fig. 2H). NM-spheroids showed higher expression of the MSC marker *VCAM-1* (Fig. 2I). With regard to stem cell markers, HpMSC-NM-spheroids maintained *SOX2* and *OCT3/4* expression at the same level as LpMSC-NM-spheroids, whereas expression of these genes decreased in Hp-Adh-MSCs (Fig. 2J).

### Immunomodulatory Property of NM-spheroids

**Figure 3.**
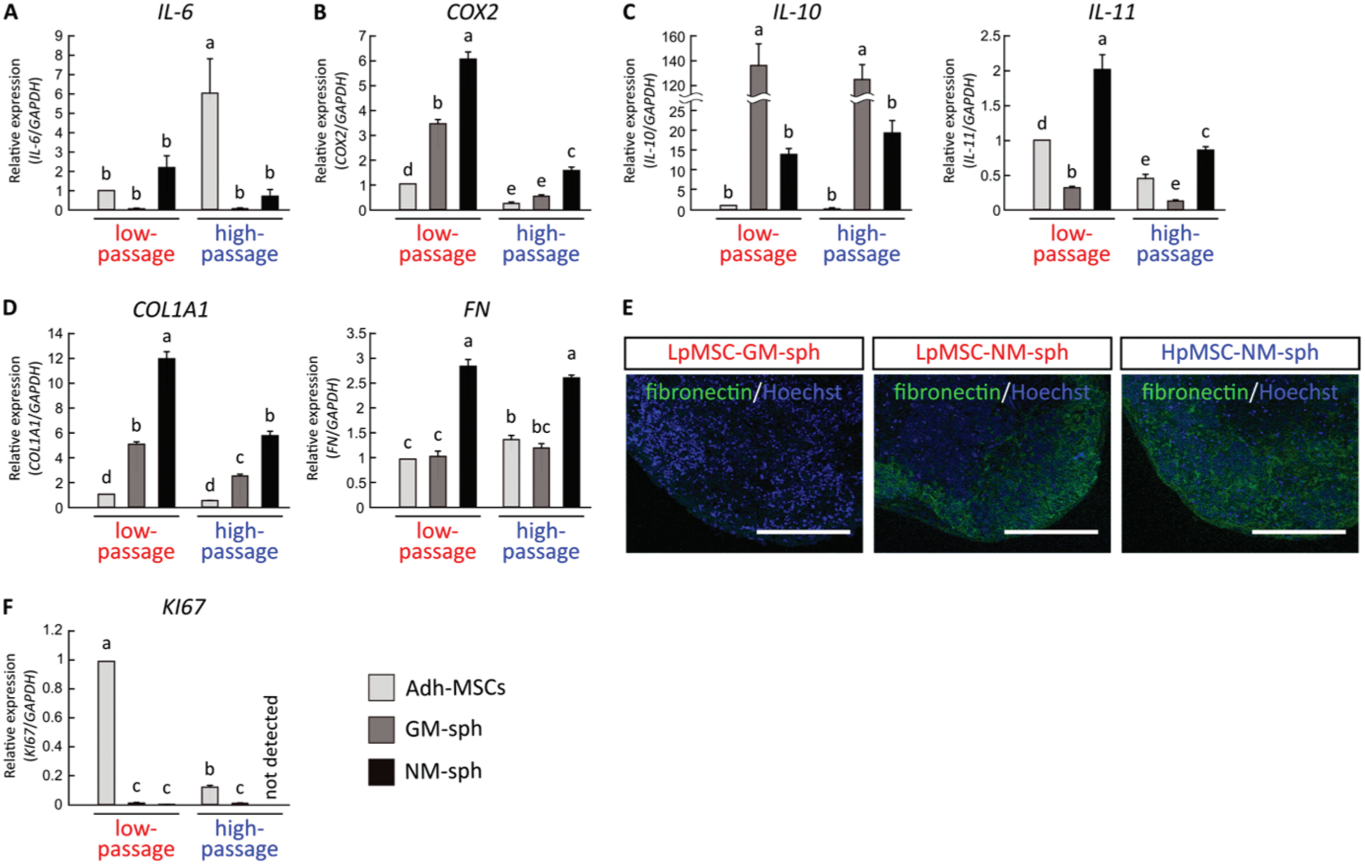
Immunomodulatory marker expression and ECM components of NM-spheroids.(A-C) Relative expression of (A) pro-inflammatory marker (*IL-6*), (B) immunomodulatory marker (*COX2*), and (C) anti-inflammatory markers (*IL-10*, *IL-11*) determined by real-time RT-PCR. (D) Relative expression of ECM markers (*COL1A1*, *FN*) determined by real-time RT-PCR. (E) Immunocytochemistry for fibronectin. Cells were counterstained with Hoechst to reveal the nuclei. Scale bars = 200 µm. (F) Relative expression of cell proliferation marker (*KI67*) determined by real-time RT-PCR. *GAPDH* expression was used as an internal control (mean ± SD, *n* = 3; different letters indicate significant differences, *P* < .05, ANOVA with Tukey’s multiple comparison test). Adh-MSCs: Adherent MSCs, GM-sph: 3D spheroids cultured with GM, NM-sph: 3D spheroids cultured with NM. Abbreviations: ECM, extracellular matrix; GM, growth medium; NM, neurosphere medium; RT-PCR, reverse-transcription polymerase chain reaction.

MSC spheroids exhibit anti-inflammatory properties.^21,22^ When compared with adherent cells, Hp-Adh-MSCs showed significant upregulation of the pro-inflammatory cytokine *IL-6* and downregulation of the immunomodulatory marker *COX2* relative to Lp-Adh-MSCs (Fig. 3A, 3B). However, NM-spheroids showed significantly lower expression of *IL-6* than Hp-Adh-MSCs and upregulation of *COX2* (Fig. 3A, 3B). GM-spheroids showed the highest expression of the anti-inflammatory cytokine *IL-10*, although *IL-10* and *IL-11* expression was still higher in NM-spheroids than in adherent cells (Fig. 3C). These findings suggest that NM-spheroids exhibit improved immunomodulatory properties compared to adherent cells and GM-spheroids, which may enhance the therapeutic potential of MSCs.

### Expression of ECM in NM-spheroids

The expression of collagen type I (*COL1A1*) and fibronectin (*FN*), two of the main components of the ECM, was significantly higher in NM-spheroids than in adherent cells and GM-spheroids (Fig. 3D). Immunocytochemistry revealed a lack of *FN* expression in LpMSC-GM-spheroids, whereas both LpMSC- and HpMSC-NM-spheroids showed expression at the periphery of the spheroids, just beneath the surface cell layers (Fig. 3E). The gene expression profiles of NM-spheroids derived from donor 2 and donor 3 were similar with respect to neural crest, MSC, stem cell, ECM, and proliferation markers based on real-time PCR analysis (Supplementary Fig. S3A, S3B).

### Quiescent State of NM-spheroids

**Figure 4.**
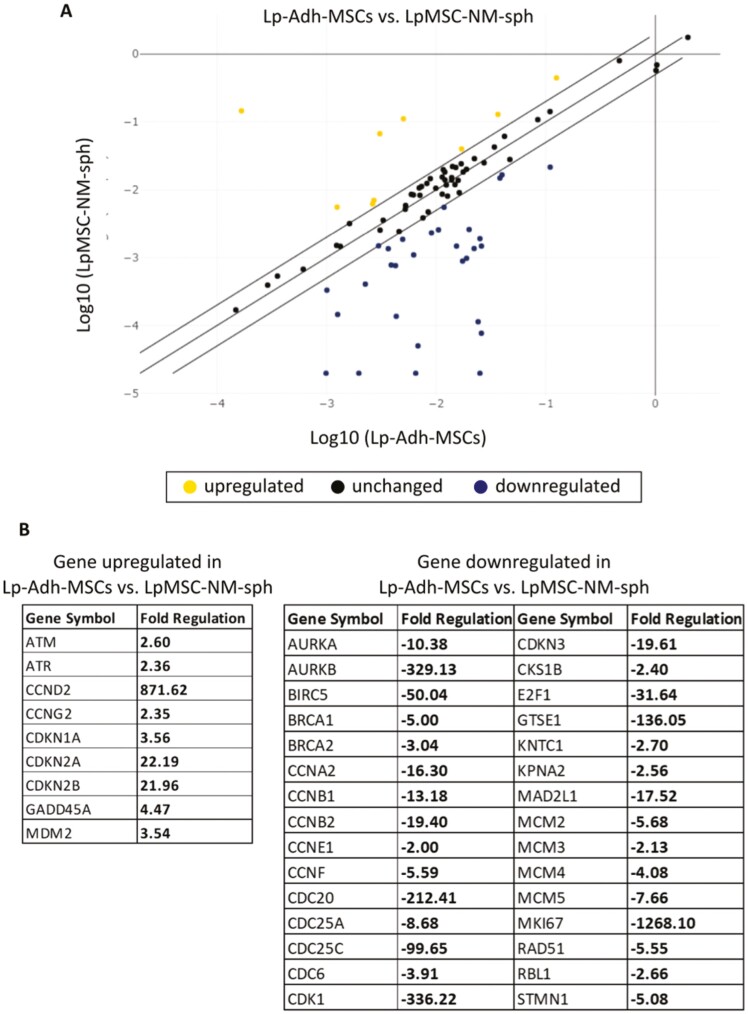
PCR array for cell cycle gene expression in NM-spheroids.(A) Scatter plot of cell cycle gene expression in LpMSC-NM-sph relative to Lp-Adh-MSCs. Genes without altered expression (black), upregulated genes (yellow), and downregulated genes (blue) are shown with threshold lines representing 2.0- and −2.0-fold changes. (B) List of genes with NM-spheroids with more than 2.0-fold upregulation or downregulation in LpMSC-NM-sph compared with Lp-Adh-MSCs. Abbreviations: MSCs, mesenchymal stem cells; NM, neurosphere medium.

GM-spheroids and NM-spheroids showed significantly reduced expression of *KI67*, a proliferation marker (Fig. 3F). As NM-spheroids did not multiply during 3 weeks of shaking (Supplementary Fig. S2C), the cells may have stopped proliferating. Stem cells reside in a quiescent state within the in vivo environment, which enables proper maintenance of stem cell characteristics. To investigate further, we performed a PCR assay of cell cycle-related gene expression to evaluate the quiescent state of NM-spheroids. In total, 39 of 84 examined genes showed more than 2.0-fold upregulation or downregulation in LpMSC-NM-spheroids compared to Lp-Adh-MSCs (Fig. 4A). In particular, genes related to cell cycle checkpoint arrest (*ATM*, *ATR*) and negative regulators of the cell cycle (cyclin-dependent kinase inhibitor, *CDKN1A*, *CDKN2A*, and *CDKN2B*) were upregulated in LpMSC-NM-spheroids (Fig. 4B). In contrast, genes related to cell division (*CCNA2*, *CCNB1*, *CCNB2*) and DNA replication (*MCM2*, *MSC3*, *MCM4*, *MCM5*) were downregulated in LpMSC-NM-spheroids compared with Lp-Adh-MSCs. These data suggest that NM-spheroids reside in a quiescent state.

### Effect of NM on Adherent MSCs

NM is typically used to form spheroids from neural crest-derived tissues,^26^ but the effect of NM on adherent cultured BM-MSCs is unknown. We cultured Lp-Adh-MSCs and Hp-Adh-MSCs with NM for 3 days in adherent culture conditions. Both Lp-Adh-MSCs and Hp-Adh-MSCs cultured with GM reached 70%-80% confluence and showed a spindle-shaped morphology (Supplementary Fig. S4A, left). However, adherent MSCs with NM showed low proliferation compared with adherent MSCs with GM (Supplementary Fig. S4A, S4B). Both Lp-Adh-MSCs with NM and Hp-Adh-MSCs with NM showed high expression of neural crest (*SOX9*, *SLUG*, *NES*), MSC (*VCAM-1*), stem cell (*SOX2*, *OCT3/4*), and ECM markers (*FN*) (Supplementary Fig. S4C). Adherent MSCs with NM showed low expression of a cell proliferation marker (*KI67*) with reduced cell growth ability, which agrees with the DNA quantification data (Supplementary Fig. S4B, S4C). These data suggest that NM itself upregulates MSC-related gene expression in MSCs in a quiescent state compared to GM medium. However, it was difficult to harvest a sufficient amount of MSCs for further experiments. NM-spheroids are expected to provide a simple way to obtain undifferentiated cells with an enhanced stem cell phenotype.

### Bone Regenerative/Remodeling Effect of NM-spheroids in Rat Bone Defects

**Figure 5.**
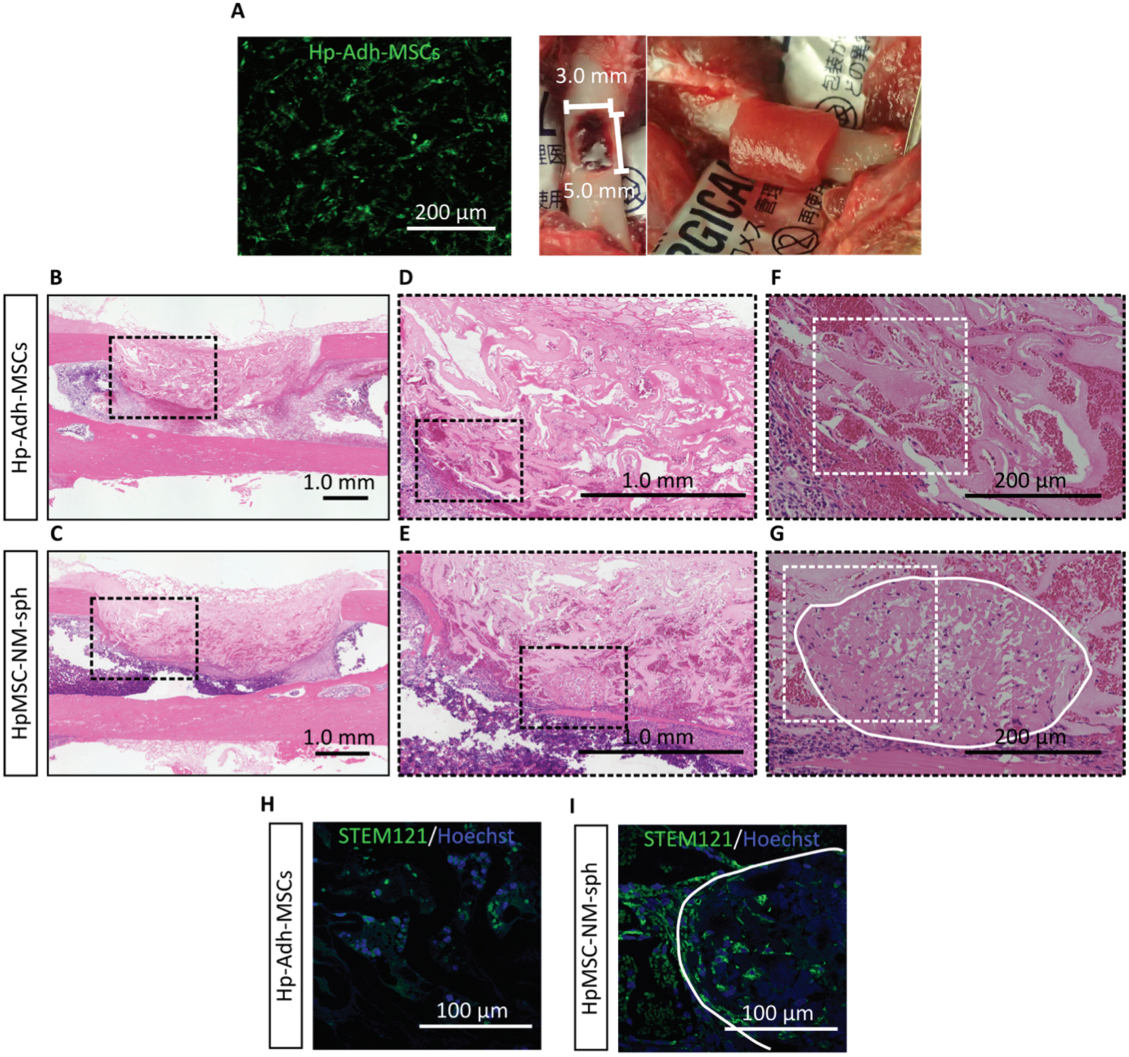
Cell engraftment of NM-spheroids in a rat femur bone defect model. (A) Left panel indicates the existence of abundant fluorescent-labeled MSCs in the collagen sponge after 12 hours of seeding. Middle panel shows the rat critical-sized femur bone defect model with 5- × 3-mm defect. Right panel shows local transplantation of the collagen sponge loaded with human BM-MSCs. (B-G) Representative light microscope images of histological cross-sections with H&E staining at the long midline of the rat femur defect model 3 days after surgery. Black dash-enclosed regions in the low-magnification images indicate the region shown in the highly magnified images. White dash-enclosed regions indicate the region shown in the immunocytochemistry images. White line indicates the outline of the NM-spheroids. (H, I) Immunocytochemistry for human cell marker STEM121. Cells were counterstained with Hoechst to reveal the nuclei. White line indicates the outline of the NM-spheroids. Experiments were performed using 3 samples per group with similar results in each group for B-I. Abbreviations: BM-MSCs, bone marrow-derived mesenchymal stem cells; NM, neurosphere medium.

To determine the therapeutic potential of NM-spheroids in bone repair, MSCs were transplanted into rat femur bone defects. To this end, Hp-Adh-MSCs or HpMSC-NM-spheroids were incorporated into a collagen sponge and implanted into a large bone defect in the rat femur (Fig. 5A). The transplanted Hp-Adh-MSCs were labeled using a cell tracer, which showed the existence of abundant cells in the collagen sponge after 12 hours of seeding (Fig. 5A). H&E staining at 3 days after surgery showed that the collagen vehicle remained in the bone defect and was situated from the cortical defect to the center of the bone marrow (Fig. 5B, 5C). Cells were diffused throughout the collagen vehicle in the Hp-Adh-MSC-implanted samples (Fig. 5D, 5F). Transplanted cells, identified by the human cell marker STEM121, were widely and sparsely distributed in the Hp-Adh-MSC-implanted collagen vehicle (Fig. 5H). In contrast, the 3D structure of spheroids was observed in sites implanted with HpMSC-NM-spheroids (Fig. 5E, 5G), and cells that migrated out from spheroids existed around the spheroids at high concentration (Fig. 5I).

**Figure 6.**
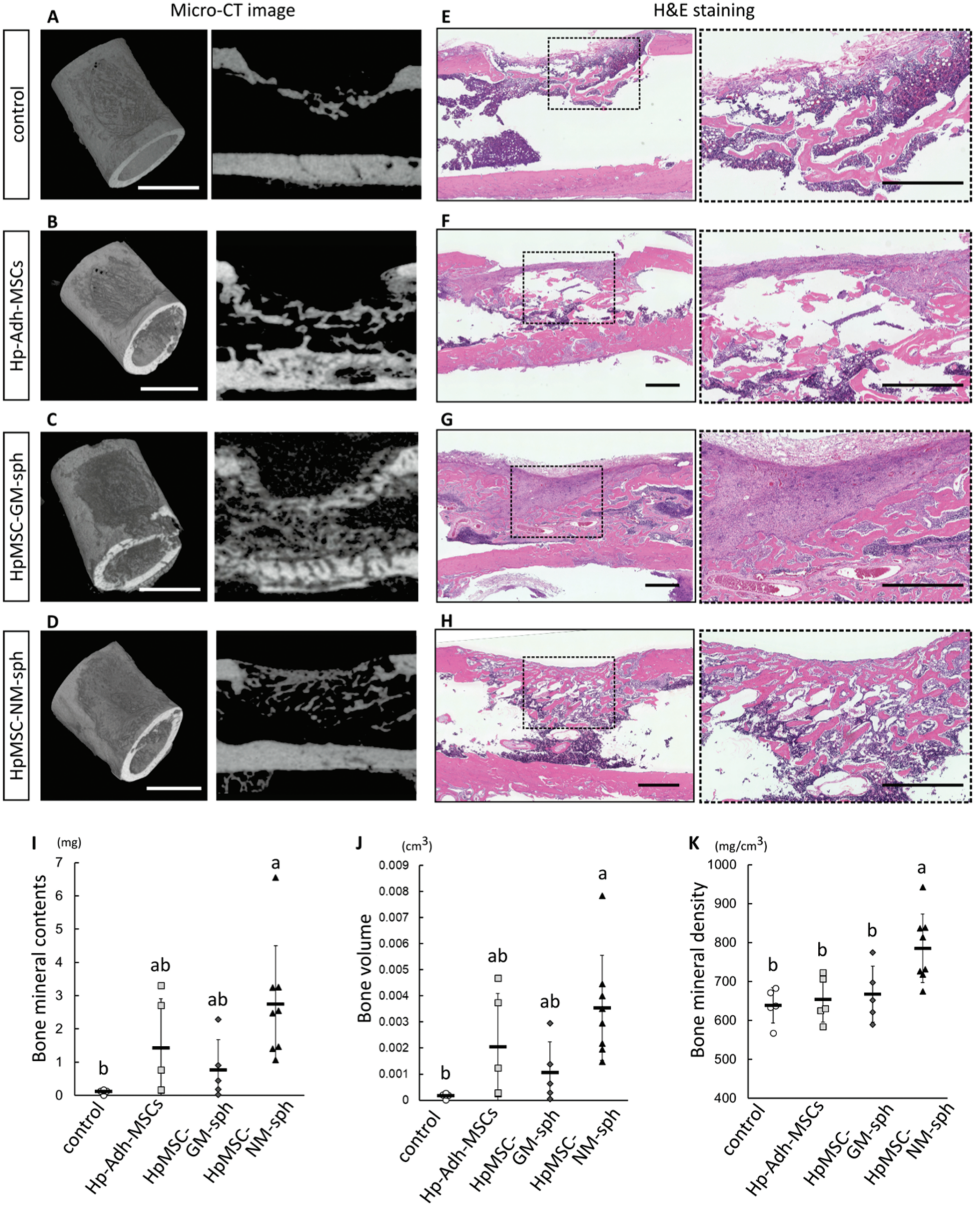
Bone remodeling/regenerative effect of NM-spheroids in a rat femur bone defect model. (A-D) Representative micro-CT images of the rat femur defect model 3 weeks after surgery. The images on the left are an overhead view of the constructed 3D bone architecture after setting a threshold for the bone mineral density based on a graded hydroxyapatite phantom. The images on the right show the original grayscale cross-section at the long midline of the defect. Scale bars = 3.0 mm. (E-H) Representative light microscope images of histological cross-sections with H&E staining at the long midline of the rat femur defect model. Black dash-enclosed regions in the images with the lowest magnification indicate the region shown in the highly magnified images. Scale bars = 1.0 mm. (I-K) Quantitative assessment of 3D bone morphometrical parameters. Bone mineral content, bone volume, and bone mineral density measurements were collected for the areas of interest in the defect regions (mean ± SD, *n* = 4-8; different letters indicate significant differences, *P* < .05, ANOVA with Tukey’s multiple comparison test). Hp-Adh-MSCs: high-passage adherent MSCs, HpMSC-GM-sph: Hp-Ad-MSC-derived GM-spheroids, HpMSC-NM-sph: Hp-Ad-MSC-derived NM-spheroids. Abbreviations: ANOVA, analysis of variance; GM, growth medium; micro-CT, micro-computed tomography; NM, neurosphere medium; NM, neurosphere medium.

To investigate the bone regeneration ability, Hp-Adh-MSCs, HpMSC-GM-spheroids, and HpMSC-NM-spheroids were prepared for transplantation into rat femur bone defects. After 3 weeks of healing, x-ray images of control samples without cells or samples transplanted with Hp-Adh-MSCs and HpMSC-GM-spheroids indicated a thin and disconnected mineralized structure in the bone marrow region (Fig. 6A-6C). New bone formation in control samples was observed beneath the bone marrow space (Fig. 6E). Intensive bone formation was observed in the bone marrow region, with fibrillary connective tissue filling the defect regions implanted with Hp-Adh-MSCs and HpMSC-GM-spheroids implanted samples (Fig. 6F, 6G). In contrast, a thick and contiguous mineralized structure was observed in the defects implanted with HpMSC-NM-spheroids, where the defect was nearly completely closed (Fig. 6D). The defects implanted with HpMSC-NM-spheroids were mostly closed with compacted and contiguous bone structures (Fig. 6H). The average bone mineral density and bone volume in the defect region were significantly higher in the sites implanted with HpMSC-NM-spheroids than in the control (mean ± SD, *n* = 4-8, *P* < .05, Fig. 6I, 6J). In particular, the bone mineral density in the defect region was significantly higher for defects implanted with HpMSC-NM-spheroids than in other samples (mean ± SD, *n* = 4-8, *P* < .05, Fig. 6K). When transplanted STEM121-labeled cells were evaluated at 3-week post-surgery, few or no fluorescent protein-positive cells were detected in both Hp-Adh-MSC and HpMSC-GM-spheroid samples (data not shown).

**Figure 7.**
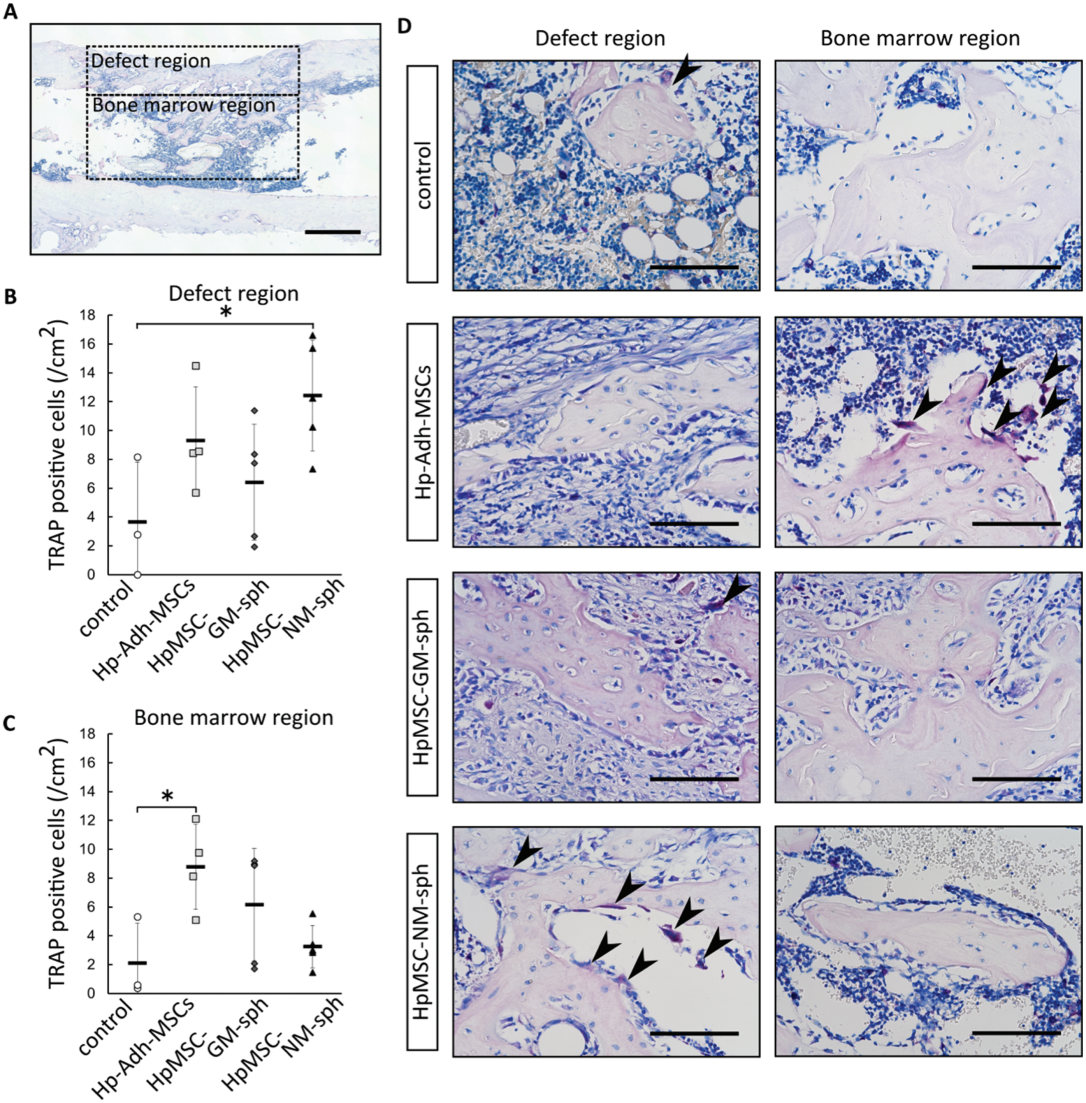
TRAP staining of the rat femur bone defect model. (A) Representative light microscope images with TRAP staining. Black dash-enclosed regions indicate the defect region and the bone marrow region. Scale bars = 1.0 mm. (B, C) Number of osteoclasts in the defect region and bone marrow region (mean ± SD, *n* = 3-5; *P* < .05, ANOVA with Dunnett’s multiple comparison test, comparison to control). (D) Representative highly magnified images of TRAP staining. Arrows point to osteoclasts identified as TRAP-positive multinuclear cells in the defect region and the bone marrow region. Scale bars = 100 µm. Hp-Adh-MSCs: high-passage adherent MSCs, HpMSC-GM-sph: Hp-Adh-MSCs-derived GM-spheroids, HpMSC-NM-sph: Hp-Adh-MSCs-derived NM-spheroids. Abbreviations: ANOVA, analysis of variance; GM, growth medium; MSCs, mesenchymal stem cells; NM, neurosphere medium; TRAP, tartrate-resistant acid phosphatase.

Tartrate-resistant acid phosphatase (TRAP) staining was performed to reveal bone remodeling and identified osteoclasts in the cortical bone defect and bone marrow region (Fig. 7A). TRAP^+^ cells were observed around the regenerated bone, aligned on the surface. The defects in the control samples showed a lower number of osteoclasts, whereas defects implanted with cells showed a large number of osteoclasts (Fig. 7B). Moreover, high amounts of osteoclasts were detected in the cortical bone defect regions implanted with HpMSC-NM-spheroids (mean ± SD, *n* = 3-5, *P* < .05, Fig. 7B, 7D). In contrast, osteoclasts were spread throughout the bone marrow region in defects implanted with Hp-Adh-MSCs (mean ± SD, *n* = 3-5, *P* < .05, Fig. 7C, 7D). These results suggest that NM-spheroids could facilitate bone regeneration and support physiological bone remodeling.

## Discussion

In this study, we successfully established spheroids in neurosphere culture conditions using the shaking culture method. Circular-shaped NM-spheroids formed with high cell viability, similarly to GM-spheroids.^25^ Bellotti et al reported a long-term analysis of MSC spheroids and found that the necrotic area in the spheroids increased after 2 weeks of static culture.^34^ Dynamic conditions have been reported to improve the viability of cells in the center of other tissue constructs cultured in vitro by enhancing mass transport to the interior of the constructs and facilitating removal of metabolic waste products from the cells,^35,36^ which may explain the high cell viability of the NM-spheroids with a 3-week culture.

In this study, we used MSCs enriched for PI^-^/LNGFR^+^/THY-1^+^ cells, which show cellular homogeneity with robust multi-lineage differentiation and self-renewal potency.^10^ Indeed, these MSCs showed high proliferation and differentiation even at 5-9 passages (Fig. 1), indicating that these cells maintained their MSC phenotype. Therefore, we defined the MSCs with 5-9 passages as LpMSCs in this study for the purpose of comparison with HpMSCs (15-18 passages), which had an impaired MSC phenotype. Using this shaking culture method, we also attempted to generate MSC spheroids directly from bone marrow cells without enrichment; however, spheroid formation hardly occurred (data not shown). We assume that the MSC spheroids obtained by this shaking culture method are composed of a relatively homogeneous stem cell population. It is likely that such a heterogeneous bone marrow cell population would be insufficient to provide enough MSCs to generate spheroids stably.

Interestingly, no significant differences were observed between LpMSC- and HpMSC-NM-spheroids in terms of spheroid size, histology, and differentiation ability. We previously reported that the size of both LpMSC- and HpMSC-GM-spheroids generated using this shaking culture method hardly increased past 1500 µm in Feret’s diameter when cultures for up to 2 months.^25^ We assume that mechanical stimulation by the shaking culture condition might lead to stem cell quiescence^37^ in spheroids by generating a specific niche environment along with enhanced uptake and diffusion of gases and nutrients, thereby preventing overgrowth of the spheroids.^25^ Thus, the shaking culture using NM in this study also maintained the size of most spheroids at less than 1500 µm. We believe that this unique characteristic of preventing spheroid overgrowth could be an advantage of this shaking culture technique for keeping spheroid cells alive. Indeed, although the method intrinsically limited spheroid growth, a few spheroids grew had grown beyond 1500 µm by 3 weeks, and necrotic cells were observed at the central zone in these spheroids.

MSC-like spindle-shaped cells that migrated from the NM-spheroids showed expansion on plastic plates with multi-lineage differentiation potential toward mesenchymal and neural crest lineages. These results suggest that the original differentiation ability of MSCs did not diminish even when cultured in NM. Moreover, HpMSC-NM-spheroid culture restored the multipotency of Hp-Adh-MSCs, which had impaired differentiation potential prior to spheroid culture. The neural crest markers *SOX9*, *SLUG*, and *NES*; MSC marker *VCAM-1*; and stem cell markers *SOX2* and *OCT3/4* were highly expressed in LpMSC- and HpMSC-NM-spheroids (Fig. 2H-2J). As the adherent MSCs cultured in NM showed upregulation of these genes (Supplementary Fig. S4C), not only 3D aggregation but also the components of the NM itself affected the NCSC/MSC-related gene profiles. MSCs in an undifferentiated state have been reported to express neural crest marker genes, and these genes are upregulated in neurosphere formation.^29,38^ In particular, nestin is downregulated during osteogenic differentiation in MSCs, suggesting that it is a marker of undifferentiated MSCs.^39^ Thus, high expression of neural crest markers is suggested to enhance both the MSC and NCSC characteristics of NM-spheroids compared to adherent cells and GM-spheroids. It should be noted that SOX2 is not only the key factor of stemness but also an important regulator of NCSCs.^40^ The NM used in this study is commonly used to isolate and maintain NCSCs. Although speculative, particular components in the NM, such as EGF, N-2, and B27, might have preferentially stimulated the expression of the representative NCSC markers *SOX2* and *NES* in the MSC spheroids (Fig. 2H, 2J) to obtain a NCSC-like phenotype.

The immunomodulatory function of MSCs is an important factor in tissue engineering. MSCs have been found to interact directly with immune cells or secrete soluble factors, allowing them to influence neighboring cells and regulate the immune environment.^41^ For example, IL-10 secreted from MSCs suppresses T-cell proliferation^42^ and influences Tregs by enhancing their immunosuppressive capacity.^43^ COX2 is a stress-responsive gene and a key enzyme in the production of prostaglandins during inflammation; it is also critically involved in bone healing through its regulation of MSC differentiation.^44^ MSCs themselves express COX2 and produce prostaglandin, which regulates the immunosuppressive properties of MSCs.^17^ It has been reported that the formation of 3D spheroids from MSCs enhances their anti-inflammatory properties, even when the spheroids are generated from expanded MSCs.^21,22,45^ Indeed, in the present study, both LpMSC-NM-spheroids and HpMSC-NM-spheroids showed low expression of pro-inflammatory markers and high expression of immunomodulatory and anti-inflammatory markers (Fig. 3A-3C). These data suggest enhanced immunomodulation properties of NM-spheroids.

Furthermore, the critical role of the ECM in 3D aggregates has been demonstrated.^46^ It is well known that the ECM contributes to various cell functions, such as cell adhesion, proliferation, differentiation, and death.^47,48^ Significantly increased expression of ECM molecules, including *FN*, laminin, and *COL1A1*, have been observed in 3D-cultured MSCs compared to monolayer MSCs.^49^ Interestingly, ECM-enriched spheroids showed significantly higher expression of the stem cell markers *SOX2*, *OCT3/4*, and *NANOG* than ECM-poor spheroids.^20,50^ In natural tissues, stem cells interact with supporting cells and ECM by forming a niche microenvironment, which maintains cell stemness.^12,51^ Therefore, 3D-spheroids enriched in ECM are suggested to efficiently induce stemness in MSCs, most likely by mimicking the native environment. Our NM-spheroids expressed the ECM markers *COL1A1* and *FN* at higher levels compared to adherent cells and GM-spheroids, and this expression was positively correlated with the expression of the stem cell markers *SOX2* and *OCT3/4* (Fig. 3D, 3E). Although the relationship between altered gene expression and MSC function is yet to be determined, the expression of pluripotent cell-specific factors, such as *SOX2* and *OCT3/4*, appears to be essential for maintaining the proliferation and differentiation of MSCs.^52,53^ Moreover, neither GM-spheroids nor NM-spheroids multiplied after prolonged shaking culture, and they showed low expression of the proliferation marker *KI67* (Fig. 3F). Genes related to cell division and DNA replication were also downregulated in NM-spheroid cells (Fig. 4). Stem cells reside in a quiescent state in the natural environment, which enables the maintenance of stem cell function.^37^ A previous study successfully generated BM-MSCs in a quiescent state under suspension culture, which enhanced their self-renewal and differentiation potential upon reactivation.^54^ These results suggest that NM-spheroids generated by the modified neurosphere technique would provide a more suitable environment to induce the quiescent state of MSCs, which enhances their stem cell phenotype.

MSC spheroids exhibit increased paracrine secretion, proliferation, stemness, and anti-inflammatory properties, which enhances their therapeutic potential in clinical applications.^46^ Moreover, MSC spheroids can be used without scaffolds, which can prevent problems related to the use of artificial materials. However, to compare the in vivo function of adherent MSCs and spheroids in the present study, we used a collagen sponge to transplant cells as previously described for a rat femur bone defect model.^31,55^ It has been reported that the number of transplanted MSCs is positively correlated with new bone formation.^56^ Thus, MSCs often require expansion to large numbers in vitro prior to in vivo transplantation. To simulate this clinical requirement, we applied expanded MSCs and spheroids in this study.

We mainly used representative MSCs from donor 1 in the transplantation experiments because the MSCs used in this study were enriched LNGFR^+^/THY-1^+^ BM-MSCs, which have been reported to be a homogeneous population with similar stem cell properties among different donors.^10^ It should be noted that our in vitro results showed similar trends for upregulation of neural crest, MSC, stem cell, ECM, and proliferation marker genes among NM-spheroids from different donors (Fig. 2H-2J: donor 1, Supplementary Fig. S3A, S3B: donor 2 and 3). These results suggest that NM-spheroid formation equivalently improved the stem cell characteristics at 3 weeks, which would overcome small variations in cellular proliferation and senescence in the original adherent MSCs derived from different donors.

Hp-Adh-MSCs, HpMSC-GM-spheroids, and HpMSC-NM-spheroids were embedded in collagen sponges and implanted into rat critical-sized femur defects. We used SD rats with cyclosporine treatment because if immunocompromised nude rats were used in this model, their thinner femurs would fracture too easily. There was no considerable effect of cyclosporine on bone healing in the control group (without cell transplantation). After 3 weeks of healing, transplantation of HpMSC-NM-spheroids induced rapid bone regeneration with extensive bone remodeling. Similar results were obtained when Hp-Adh-MSCs and HpMSC-NM-sph from donor 2 were used (data not shown). There are several potential mechanisms underlying the contribution of NM-spheroids to bone regeneration. It has been reported that poor survival of transplanted MSCs is related to cell death and immune rejection, and that this cell loss is a critical factor underlying insufficient bone regeneration.^57^ In contrast, MSC spheroids have shown improved cell survival and engraftment in various in vivo models through upregulation of anti-apoptotic signals.^46,58,59^ These findings may explain our data showing that collagen sponges with HpMSC-NM-spheroids enabled effective engraftment of MSCs at the transplantation site, providing many migrated cells around the spheroids to facilitate bone regeneration. In contrast, Hp-Adh-MSCs transplanted as single cells were sparsely diffused; therefore, the engraftment of transplanted cells and bone formation might be limited compared to that with spheroid transplants.

Of note, we transplanted undifferentiated MSC spheroids in this study. A previous study using naïve MSCs in a rat femur defect model demonstrated that the transplanted cells aligned along the surface of the newly formed bone, suggesting an indirect contribution to bone regeneration.^31^ Therefore, NM-spheroids may regulate the surrounding cells rather than directly differentiating into osteoblasts and generating new bone tissue. MSCs have been reported to play an important role in bone regeneration by regulating various cell types.^60^ MSCs interact with endothelial cells or release exosomes that promote angiogenesis,^61^ and they affect various immune cells and regulate the immune microenvironment during tissue regeneration.^60^ For example, injection of MSCs induces apoptosis in T cells,^62^ whereas activated Tregs suppress pro-inflammatory cytokine levels and have a positive impact on MSC-mediated bone regeneration.^63^

The combination of MSCs and MSC-derived ECM has been reported to facilitate bone regeneration, whereas only MSCs or MSC-derived ECM administered alone failed to enhance bone regeneration.^64,65^ The NM-spheroids obtained in the present study showed significant upregulation of VCAM-1, an adhesion molecule involved in blood vessel maturation.^58^ Moreover, NM-spheroids had high stem cell marker expression, enhanced immunomodulatory properties, and enriched ECM expression. These unique properties were maintained even after 3 weeks of prolonged shaking culture.

In this study, the transplanted NM-spheroids had almost disappeared at 3 weeks after transplantation. Upon human MSC transplantation, the number of MSCs was previously shown to dramatically decrease from 2 to 4 weeks during the bone regeneration process.^64^ The favorable bone regeneration even with short-term engraftment of implanted MSCs is partly explained by the paracrine function of apoptotic MSCs, which supports osteoclastogenesis.^66^ In an ectopic bone formation model, human MSCs were observed to disappear from the implantation site after 2 weeks, but the MSCs still showed immunomodulatory properties by inducing differentiation of circulating hematopoietic stem cells into osteoclasts, leading to bone formation.^67^ These previous reports may explain our finding that NM-spheroids enhanced bone regeneration along with the appearance of many osteoclasts at 3 weeks even though the transplanted MSCs disappeared, possibly by apoptosis. It is difficult to attribute the in vivo bone formation ability to the mechanisms observed in vitro. However, the enhanced stem cell characteristics and unique composition of NM-spheroids may facilitate bone healing in vivo. Future research should address the biological mechanism of NM-spheroid-mediated bone formation.

## Conclusions

A 3D shaking culture method, in which NM was used, generated NM-spheroids with enhanced stem cell characteristics by maintaining and restoring the multipotency of the cells as well as upregulating MSC-related genes and immunomodulatory genes in vitro. The unique characteristics of NM-spheroids facilitate enhanced bone regeneration upon local transplantation, which should have great clinical potential for bone and tissue regenerative therapies.

## Supplementary Material

szab030_suppl_Supplementary_Material

## Data Availability

The data that support the findings of this study are available from the corresponding authors, Kunimichi Niibe or Hiroshi Egusa, upon reasonable request.
