# Supplementary material for: Novel Mesenchymal Stem Cell Spheroids with Enhanced Stem Cell Characteristics and Bone Regeneration Ability
Source: Stem Cells Transl Med. 2022 Mar 10;11(4):434–49. doi: 10.1093/stcltm/szab030 (PMC9052431; doi:10.1093/stcltm/szab030)
Supplement: szab030_suppl_Supplementary_Material [file szab030_suppl_supplementary_material.docx]

**Supplementary information**

**Supplementary Figure 1**

**
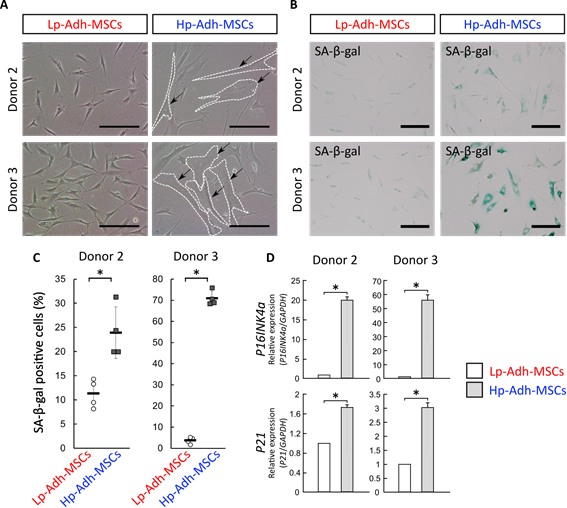
**

**Fig. S1. Cellular senescence of human BM-MSCs after long-term adherent culture.**

(A) Phase contrast images of low-passage adherent MSCs (Lp-Adh-MSCs) and high-passage adherent MSCs (Hp-Adh-MSCs) (donors 2 and 3). Notes: Arrowhead: location of enlarged cells; dotted line: outline of enlarged cells. Scale bars: 200 µm. (B) SA-β-gal assay of Lp-Adh-MSCs and Hp-Adh-MSCs (donors 2 and 3). Scale bars: 200 µm. (C) Quantitative analysis of SA-β-gal-positive cells (donors 2 and 3) (mean ± SD, n=4; *P*<0.05, Student’s *t*-test). (D) Relative expression of *P16INK4a* and *P21* determined by real-time RT-PCR (mean ± SD, n=3; *P*<0.05, n.s, not significant, Student’s *t*-test).

**
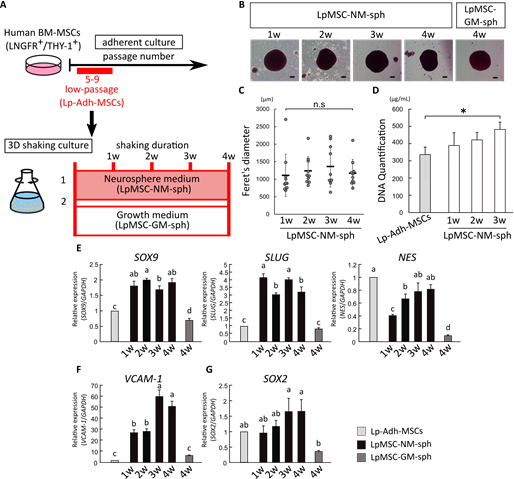
Supplementary Figure 2**

**Fig. S2. Optimization of MSC spheroids with neurosphere medium under 3-D shaking culture.**

(A) Schema of a 3-D shaking culture system containing low-passage adherent MSCs (Lp-Adh-MSCs). (B) Phase contrast images of Lp-Adh-MSCs-derived NM-spheroids (LpMSC-NM-sph) and Lp-Adh-MSCs-derived GM-spheroids (LpMSC-GM-sph). Scale bars: 200 µm. (C) Feret’s diameter of LpMSC-NM-sph subjected to different shaking durations (mean ± SD, n=10; n.s, not significant, ANOVA with Tukey’s multiple comparison test). (D) DNA quantification analysis of Lp-Adh-MSCs and LpMSC-NM-sph (mean ± SD, n=3; *P*<0.05, ANOVA with Tukey’s multiple comparison test). (E-G) Relative expression of (E) neural crest markers (*SOX9*, *SLUG*, *NES*), (F) MSC marker (*VCAM-1*) and (G) stem cell marker (*SOX2*) determined by real-time RT-PCR. *GAPDH* expression was used as an internal control (mean ± SD, n=3; different alphabets indicate significant differences, *P*<0.05, ANOVA with Tukey’s multiple comparison test). Lp-Adh-MSCs: low-passage adherent MSCs, LpMSC-NM-sph: Lp-Adh-MSC-derived NM-spheroids, LpMSC-GM-sph: Lp-Adh-MSC-derived GM-spheroids.

**Supplementary Figure 3**

**
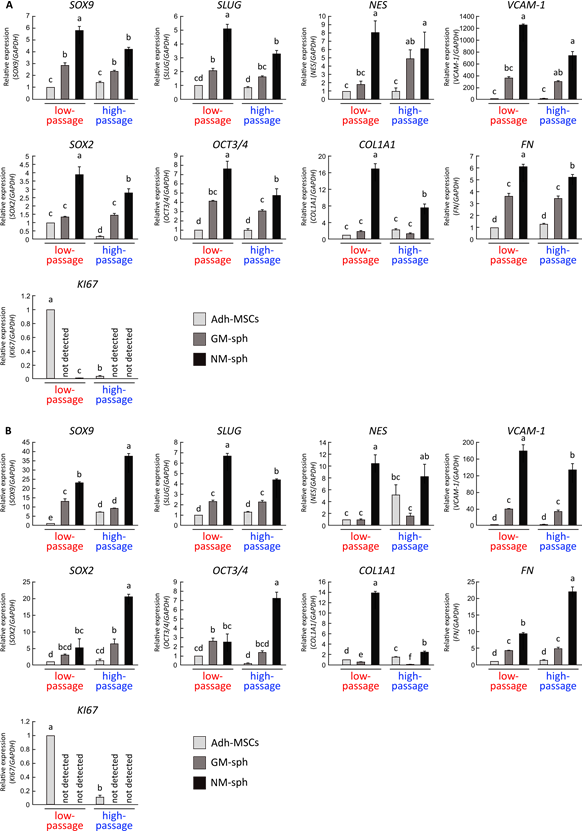
**

**Fig. S3. Gene expression profile of NM-spheroids.**

(A-B) Gene expression profile of cells from (A) donor 2 and (B) donor 3. Relative expression of neural crest markers (*SOX9*, *SLUG*, *NES*), MSC marker (*VCAM-1*), stem cell markers (*SOX2*, *OCT3/4*), ECM markers (*COL1A1*, *FN*), and cell proliferation marker (*KI67*) determined by real-time PCR. *GAPDH* expression was used as an internal control (mean ± SD, n=3; different letters indicate significant differences, *P*<0.05, ANOVA with Tukey’s multiple comparison test). Adh-MSCs: Adherent MSCs, GM-spheroids: 3-D spheroids cultured with GM, NM-spheroids: 3-D spheroids cultured with NM.

**
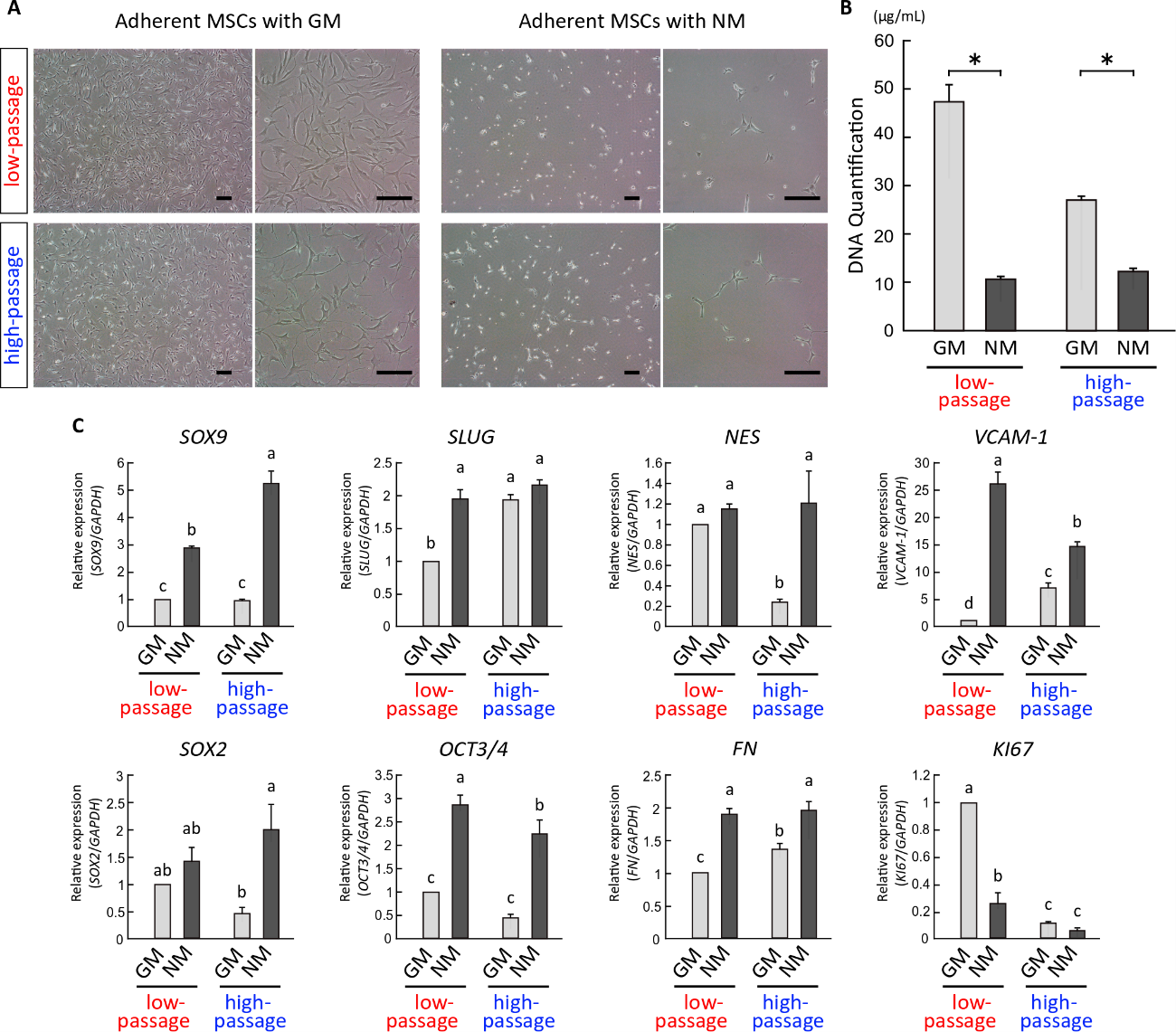
Supplementary Figure 4**

**Fig. S4. Effect of neural stem cell medium on characteristics of adherent MSCs.**

(A) Phase contrast images of adherent MSCs cultured in either GM or NM on day 3. Scale bars: 200 µm. (B) DNA quantification analysis of adherent MSCs subjected to different culture media on day 3 (mean ± SD, n=3; *P*<0.05, Student’s *t*-test). (C) Relative expression of neural crest markers (*SOX9*, *SLUG*, *NES*), MSC marker (*VCAM-1*), stem cell markers (*SOX2*, *OCT3/4*), ECM marker (*FN*), and cell proliferation marker (*KI67*) determined by real-time RT-PCR. *GAPDH* expression was used as an internal control (mean ± SD, n=3; different letters indicate significant differences, *P*<0.05, ANOVA with Tukey’s multiple comparison test). GM: Adherent MSCs cultured with GM, NM: Adherent MSCs cultured with NM.

**Supplementary Table**

**Table S1: Primers used for real-time RT-PCR**

| Gene | Primer sequence (5'-3') (forward/reverse) | Product size (bp) | Accession number |
| --- | --- | --- | --- |
| *P16INK4a* | AGCATGGAGCCTTCGGCTGA | 142 | XM_011517676.2 |
|  | CCATCATCATGACCTGGATCG |  |  |
| *P21* | GAGACTCTCAGGGTCGAAAA | 92 | NM_001291549.1 |
|  | TTAGGGCTTCCTCTTGGAGA |  |  |
| *SOX9* | CGAAATCAACGAGAAACTGGAC | 87 | NM_000346.4 |
|  | ATTTAGCACACTGATCACACG |  |  |
| *SNI2* | GCAGTGAGGGCAAGAAAAAG | 121 | NM_003068.5 |
| *(SLUG)* | TCGGACCCACACATTACCTT |  |  |
| *NESTIN* | AAGATGTCCCTCAGCCTGGA | 99 | NM_006617.2 |
| *(NES)* | GAGGGAAGTCTTGGAGCCAC |  |  |
| *VCAM-1* | CGAAAGGCCCAGTTGAAGGA | 141 | NM_001078.4 |
|  | GAGCACGAGAAGCTCAGGAGAAA |  |  |
| *SOX2* | TGGTCCTGCATCATGCTGTAG | 71 | NM_003106.4 |
|  | AACCAGCGCATGGACAGTTAC |  |  |
| *OCT4* | GCAGCGACTATGCACAACGA | 194 | NM_002701.6 |
|  | CAGAGTGGTGACGGAGACA |  |  |
| *COL1A1* | GTGCTAAAGGTGCCAATGGT | 228 | NM_000088.4 |
|  | CTCCTCGCTTTCCTTCCTCT |  |  |
| *FIBRONECTIN* | AAACTTGCATCTGGAGGCAAACCC | 146 | NM_212482.3 |
| *(FN)* | AGCTCTGATCAGCATGGACCACTT |  |  |
| *IL-6* | GGTACATCCTCGACGGCATCT | 81 | NM_001371096.1 |
|  | GTGCCTCTTTGCTGCTTTCAC |  |  |
| *COX2* | CCCTTGGGTGTCAAAGGTAA | 169 | NM_000963.4 |
|  | GCCCTCGCTTATGATCTGTC |  |  |
| *IL-10* | GGCGCTGTCATCGATTTCTT | 101 | NM_000572.3 |
|  | GGCTTTGTAGATGCCTTTCTCTTG |  |  |
| *IL-11* | TCTCTCCTGGCGGACACG | 79 | NM_000641.4 |
|  | AATCCAGGTTGTGGTCCCC |  |  |
| *KI67* | ACGCCTGGTTACTATCAAAAGG | 209 | NM_001145966.2 |
|  | CAGACCCATTTACTTGTGTTGGA |  |  |
| *GAPDH* | GAAGGTGAAGGTCGGAGTCA | 226 | NM_002046.7 |
|  | GAAGATGGTGATGGGATTTC |  |  |

**Supplementary methods**

**S1. Induction of mesenchymal lineage**

Osteogenic and adipogenic differentiation assays were initiated once adherent cultured MSCs and plated MSC spheroids reached ~60% confluence. For osteogenic induction, cells were maintained in osteogenic differentiation medium consisting of GM supplemented with 10 mM β-glycerophosphate (Sigma-Aldrich, St. Louis, MO, USA), 50 µM L-ascorbic acid (Sigma-Aldrich), and 100 nM dexamethasone (Sigma-Aldrich), and cultured for 21 days. The differentiation of the cells into osteoblasts was confirmed by ALP staining (Nichirei, Tokyo, Japan) and Alizarin Red S staining (Sigma-Aldrich). For adipogenic induction, cells were maintained in adipogenic induction medium (Lonza) and cultured for 14 days. The differentiation of the cells into adipocytes was confirmed by Oil Red O staining (Wako).

For chondrogenic induction, adherent cultured cells were harvested and 3 × 10^5^ cells were transferred into a 15-mL tube. The tube was centrifuged at 100 × *g* for 4 min at room temperature and the resulting pellet was resuspended in chondrogenic induction medium (Lonza). The tube was centrifuged again at 100 × *g* for 4 min and the resulting pellet was resuspended in chondrogenic induction medium with 10 ng/mL transforming growth factor-β3 (TGF-β3) (R&D Systems, Minneapolis, MN, USA) and 500 ng/mL bone morphogenetic protein-6 (BMP6) (R&D Systems). The tube was centrifuged again at 150 *× g* for 4 min at room temperature to generate cell pellets. MSC spheroids were transferred into a 15-mL tube and maintained in chondrogenic induction medium with 10 ng/mL TGF-β3 and 500 ng/mL BMP6 and cultured for 21 days. Differentiation of the cells into chondrocytes was confirmed by Toluidine blue staining (Wako).

**S2. Induction of neural crest lineage**

Adherent cultured cells were seeded at 1.5 × 10^5^ cells/mL on non-treated 6-well culture plates (Thermo-Fisher Scientific) with NM to form neurospheres. Cell clumps were cultured for 14 days with medium exchange every 3–4 days. For neural differentiation, cell clumps and MSC spheroids were plated on poly-L-ornithine (Wako)/fibronectin (Wako)-coated 8-well chambers (Matsunami) using induction medium containing advanced DMEM supplemented with 10% FBS, 1% N-2 supplement, 1% penicillin-streptomycin, and 10 mM HEPES. The medium was changed every 3–4 days until day 10. For immunocytochemistry of differentiated neural lineage cells, the cells were fixed in 4% PFA (Wako) for 30 min, and permeabilized with 0.3% Triton X-100 (Wako) for 5 min. After blocking in 10% FBS buffer for 30 min at room temperature, the cells were incubated overnight at 4 °C with the following primary antibodies: rabbit polyclonal anti-βIII tubulin (1:2000 dilution; Abcam, Cambridge, UK) and mouse monoclonal anti-αSMA (1:200; Sigma-Aldrich). The cells were incubated for 1 h at room temperature with the following secondary antibodies: anti-rabbit IgG (1:500; Invitrogen Abcam MA, USA), anti-mouse IgG (Alexa 488; 1:500; Abcam), and anti-mouse IgG2a (Alexa 488; 1:200; Abcam). They were mounted with Vectashield (Vector Laboratories, Burlingame, CA, USA) and observed under a confocal microscope (LSM 780; Carl Zeiss, Jena, Germany).

**S3. Cell viability of MSC spheroids**

The cell viability of the MSC spheroids was investigated using a Live/Dead Viability/Cytotoxicity Kit (Invitrogen). MSC spheroids were washed with PBS and then gently left to stand in a glass-base dish (Iwaki, Tokyo, Japan). Spheroids were stained with 2 µM Calcein AM and 4 µM ethidium homodimer-1 for 30 min at room temperature. The stained spheroids were observed under a confocal microscope (LSM 780; Carl Zeiss).

**S4. DNA quantification analysis**

The total DNA of the MSCs was measured using a DNA quantification kit (Cosmo-Bio, Tokyo, Japan) according to the manufacturer’s protocol.

In the experiment measuring the total DNA of adherent cells and MSC spheroids, 1 × 10^7^ adherent cultured cells were harvested and transferred to a micro-test tube. For the MSC spheroids, whole spheroids were collected from a culture flask and transferred to a micro-test tube. After the cells were washed twice with PBS and 500 µL of dilution buffer was added, the cells were sonicated until complete homogenization. Then, 50 µl of various concentrations of DNA standard or cell lysates was mixed with 1 mL of dilution buffer and 50 µL of color development reagent. The mixture was transferred to a 96-well black microplate (Greiner bio-one) and the fluorescence intensity was measured using a GloMax-Multi Detection System (Promega, Madison, WI, USA) with an ultraviolet (UV) filter (excitation: 365 nm; emission: 458 nm). The total amount of DNA was determined using a calibration curve based on the value of the standard solution.

In the experiment measuring the total DNA of adherent cells cultured in GW medium or NM, adherent cells were collected from a 10-cm culture dish (Greiner bio-one) on day 3 and subjected to the protocol described above.

**S5. Senescence-associated β-galactosidase (SA-β-gal) assay**

Adherent MSCs were cultured at a cell density of 1 × 10^4^ cells/mL in 12-cm culture dishes (Greiner bio-one) and maintained in GM for 1 d. The SA-β-gal assay was performed with a Senescence Detection Kit (BioVision, Mountain View, CA, USA). Senescent cells were observed under a microscope to quantitatively determine the ratio of the number of X-gal-positive cells to the total number of cells (150–300 cells).

**S6. Immunocytochemistry of spheroid fibronectin**

MSC spheroids were fixed in 4% PFA (Wako) overnight at 4 °C. After washing with PBS, the spheroids were embedded in paraffin, sectioned at a thickness of 5 µm. For immunocytochemistry, the slides were deparaffinized and enzymatic antigen retrieval was performed with 0.1% pepsin (Nacalai Tesque) in 0.5 M acetic acid (Sigma-Aldrich) by incubating at 37 °C for 45 min in a humid chamber. After blocking in 3% skim milk (BD Biosciences, Franklin Lakes, NJ, USA) for 15 min at room temperature, the cells were incubated overnight at 4 °C with a rabbit polyclonal anti-fibronectin antibody (1:200; Bioss Antibodies, Woburn, MA, USA). Then, the cells were incubated for 1 h at room temperature with a secondary antibody [anti-rabbit IgG (Alexa 488; 1:500; Abcam)] and Hoechst (1:500; Invitrogen). They were mounted with Vectashield (Vector Laboratories) and observed under a confocal microscope (LSM 780; Carl Zeiss).

**S7. Micro-computed tomography (micro-CT) analysis**

The femur samples were fixed in 10% neutral buffered formalin (Wako) at 4 °C for 3 days. The bone volume and the mineral density of the regenerated bone at the site of cell implantation were evaluated using a ScanXmater-E090 three-dimensional micro X-ray computed tomography (CT) imaging device (Comscan Tecno, Kanagawa, Japan) and TRI/3-D-BON bone structure analysis software (Ratoc System Engineering, Tokyo, Japan). The samples were X-rayed at an energy level of 80 kV/70 mA through a 1-mm-thick brass filter. The specific thresholds for bone tissue were determined by superimposing segmented images over the original grayscale X-ray images. The bone mineral content (mg) and bone mineral density (mg/cm^3^) were determined for each sample.

**S8. Histological and immunocytochemistry analysis of *in vivo* study**

To determine the transplantation efficiency, adherent cultured cells were label by 1 nM Long Term Cell Tracer 500 Green Agent (101 Bio, Palo Alto, CA, USA) and incubated for 12 hours. The labeled cells were then harvested and resuspended in GM to a concentration of 1 × 10^6^ cells/mL. Then 1 mL of cell suspension was seeded on the commercial type I collagen-based dressing sponge (Collaplug; Integra Life Sciences) and cultured for another 12 hours. The labeled cells were observed under a confocal microscope (LSM 780; Carl Zeiss)

The harvested tissues were decalcified in 10% EDTA disodium salt (EDTA 2Na) (Muto Pure Chemicals) for 4 weeks. The decalcified solution was renewed every two days. After dehydration, the samples were embedded in paraffin. Paraffin sections with a thickness of 3–5 µm were stained with hematoxylin-eosin (H&E) for morphological observation.

Immunocytochemistry for a human cytoplasmic marker was then performed to detect the transplanted cells. The slides were deparaffinized and enzymatic antigen retrieval was performed with 0.01 M citrate buffer (Wako) with 0.05% Tween 20 (Sigma-Aldrich) by incubating at 80 °C for 30 min in a decloaking chamber (NxGen; Biocare Medical, Pacheco, CA, USA). After blocking in 2% BSA (Wako) for 30 min at room temperature, the slides were incubated overnight at 4 °C with a mouse monoclonal antibody specific for a human cytoplasmic marker (STEM121; 1:200; Takara, Shiga, Japan). The samples were incubated for 1 hour at room temperature with a secondary antibody [anti-mouse IgG (Alexa 488; 1:500; Abcam)] and Hoechst (1:500; Invitrogen). They were mounted with Vectashield (Vector Laboratories) and observed under a confocal microscope (LSM 780; Carl Zeiss).

TRAP staining was performed using a TRAP/ALP stain kit (Wako) according to the manufacturer’s instructions. They were observed under an all-in-one fluorescence microscope (BZ-X800; Keyence, Osaka, Japan).
